# A meta-analysis comparing open and minimally invasive cervical tumor surgery wound infection and postoperative complications

**DOI:** 10.1186/s12893-024-02713-8

**Published:** 2024-12-23

**Authors:** Ran Song, Mingming Ma, Nana Yang, Chunfang Chen, Huan Wang, Juan Li

**Affiliations:** Department of Gynecology, Maternal & Child Health Center Of Dezhou, No. 835 Dongdi middle Avenue, Decheng District, Dezhou, China

**Keywords:** Wound infection, Laparotomy, Minimally invasive surgery, Postoperative issues, Cervical cancer

## Abstract

To evaluate the impact of open surgical care (OSC) compared to minimally invasive surgery (MIS) on the occurrence of wound infection (WI) and overall postoperative aggregate complications (POACs) in female cervical cancer (CC) patients, we conducted this meta-analysis study. A thorough examination of the literature up to March 2024 was conducted, and 1849 related studies were examined. The 44 studies that were selected included 11,631 females who had CC. The odds ratio (ORs) and the estimation using 95% confidence intervals (CIs) were used to calculate the impact of open surgical management and MIS on WI and POACs in females with CC, using dichotomous methodologies and a random or fixed model. When comparing MIS to open surgical care, there was a substantial decrease in WI (OR, 0.19; 95% CI, 0.13–0.29, *p* < 0.001) and POACs (OR, 0.49; 95% CI, 0.38–0.62, *p* < 0.001) in females with CC. On the other hand, among female patients with CC, MIS did not differ significantly from open surgical care in pelvic infection and abscess (PI&A) incidence (OR, 0.59; 95% CI, 0.31–1.16, *p* = 0.13). When compared to OSC, women with CC who underwent MIS experienced considerably fewer WI and POACs; however, there was no discernible difference in PI&A rates. However, given several of the designated examinations for the meta-analysis had relatively small sample sizes, caution must be used while handling its values.

**Supplementary Information:**

The online version contains supplementary material available at 10.1186/s12893-024-02713-8.

## Introduction

Cervical cancer (CC), including its extension, ranks as the fourth most prevalent form of cancer in females [1]. The chief choice for treating such situations was a radical hysterectomy (RH) through open surgical care (OSC) [2]. Robotic surgery, also known as laparoscopy, has gained global recognition as the most effective treatment for CC, even in its early stages, thanks to the development of the laparoscopic approach paired with minimally invasive surgery (MIS) [3]. The clinical study’s findings on laparoscopic methodology for the cervix were surprising in that they indicated poorer overall and disease-free rates of survival for MIS in 2018 compared to OSC [4]. The four National Comprehensive Cancer Network recommendations and subsequent literature referred to OSC as a typical and suggested strategy for RH for CC [5]. To fully understand the correct intent of OSC and MIS for CC, it is crucial to talk about surgical outcomes. A briefer hospital stay, a lesser amount of blood loss, and a quicker recovery time are just a few advantages that MIS has over OSC for the treatment of gynecological problems [5–8]. Like the mainstream of previous examinations, MIS offers the advantage of a 3D viewpoint and a more specific surgical setting over open surgical management [9–12]. MIS was also associated with operative difficulty, a lengthy learning curve, and a higher cost compared to OSC. There isn’t much proof to back up the superiority of any one surgical technique, and it is uncertain whether MIS is harmless and effective due to the poor quality of previous studies, the small sample sizes, and the dearth of randomized controlled trials (RCTs). There was no difference in postoperative problems between OSC and MIS, according to several previous studies on complications [13]. As instruments and methods have improved, many studies have discovered that MIS has lower POAC rates than OSC [14]. Incorrectly, it is still unknown whether female complication rates from OSC are lower than those from MIS. A chief component in the assessment of CC is one that emphasizes the gravity of the issues. In order to provide trustworthy information to compare the advantages of different surgical techniques for treating CC, our meta-analysis set out to assess the effects of open surgical interventions and MIS on wound infection (WI), POACs, pelvic infection, and abscess.

## Methods

### Eligibility criteria

The studies showing how MIS and OSC affect WI and POACs in female CC patients chosen so that an overview could be made [15]. The protocol was registered in PROSPERO (ID number: CRD2111617323).

### Information sources

**Fig. 1:**
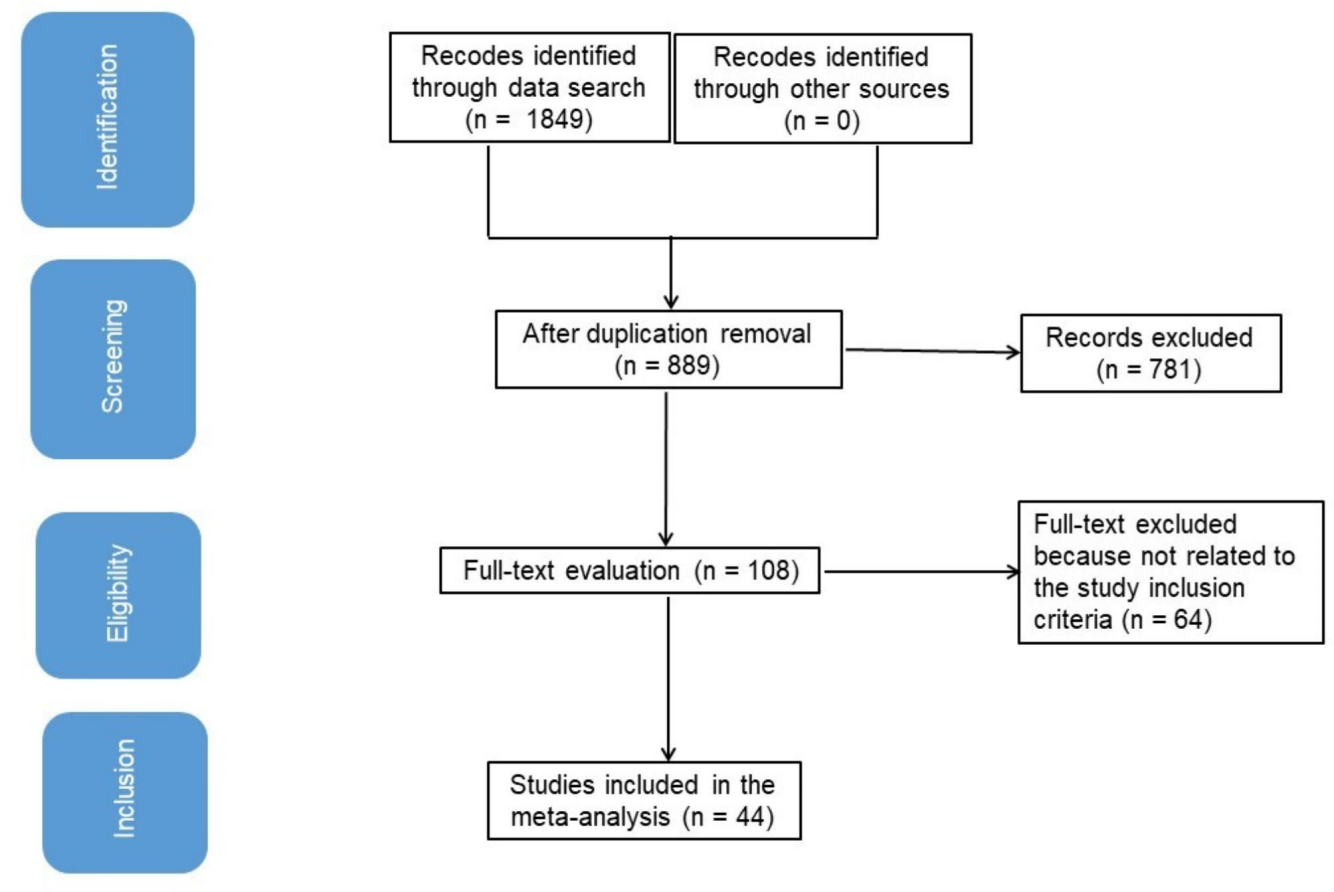
Shows the study procedure flowchart

Figure 1 is an overall study representation. When the next criteria of inclusion were satisfied, literature was incorporated into the study: [16, 17]

1. The study was a RCT, observational, retrospective, prospective one.
2. Selected female subjects for the investigation were those who had CC.
3. Open surgical management was added into the operation.
4. The study made a distinction between the impact of open surgical management and MIS on WI and POACs in CC treatment.
5. Laparoscopy studies, with or without the use of robotics, were included in the analysis.

Explorations on WIs and POACs in females without MIS and open surgical management, as well as explorations that did not examine features of consequence of MIS and open surgical management on WI and POACs in females with CC, were excluded from consideration [18, 19].

### Search strategy

We defined the search protocol operations in accordance with the PICOS opinion. WIs, POACs, pelvic infection, and abscess (PI&A) were the “outcomes”; finally, the “study design” was unrestricted. The “population” (P) consisted of female patients with CC; the “intervention” or “exposure” was open surgical management, and the “comparison” was between MIS and open surgical management [19]. In 2002, the first robotic-hysterectomy was carried out by Diaz-Arrasti [20], followed by several published trials. Hence, we searched for studies published between 2002 and until March 2024 using the following databases: the Cochrane Library, Embase, Google Scholar, PubMed, and OVID. We accomplished this by organizing keywords and connected expressions, as shown in Table 1. We eliminated paper repetitions, compiled them into an EndNote file, and reassessed their titles and abstracts to omit studies that could not demonstrate a connection between the outcomes of open surgical management and MIS, WI, and POACs in female CC patients [19–21].

**Table 1:** Search Strategy for Each Database

Database	Search strategy
**Google Scholar**	#1 “cervical cancer” OR “minimally invasive surgery” #2 “laparotomy” OR “wound infection” OR " “postoperative complication” OR “pelvic infection and abscess” #3 #1 AND #2
**Embase**	#1 ‘cervical cancer’ /exp OR ‘minimally invasive surgery’ /exp OR ‘postoperative complication’ #2 ‘laparotomy’/exp OR ‘wound infection’/exp OR ‘pelvic infection and abscess’ #3 #1 AND #2
**Cochrane library**	#1 (cervical cancer): ti, ab, kw (minimally invasive surgery): ti, ab, kw (postoperative complication): ti, ab, kw (Word variations have been searched) #2 (laparotomy): ti, ab, kw OR (wound infection): ti, ab, kw OR(pelvic infection and abscess): ti, ab, kw (Word variations have been searched) #3 #1 AND #2
**Pubmed**	#1 “cervical cancer“[MeSH] OR “minimally invasive surgery“[MeSH] OR “postoperative complication” [All Fields] #2 “laparotomy“[MeSH Terms] OR “wound infection“[MeSH] OR “pelvic infection and abscess “[All Fields] #3 #1 AND #2
**OVID**	#1 “cervical cancer“[All Fields] OR “minimally invasive surgery” [All Fields] OR “postoperative complication” [All Fields] #2 “laparotomy“[ All fields] OR “wound infection“[All Fields] or “pelvic infection and abscess“[All Fields] #3 #1 AND #2

### Selection process

A process was established after the epidemiological statement, and it was subsequently organized and scrutinized using a meta-analysis technique.

### Data collection process

The criteria used to collect data included the name of the first author, study date, study year, nation or location, population type, medical and therapeutic characteristics, categories, quantitative and qualitative estimation methods, data source, outcome estimation, and statistical analysis [22].

### Data items

When a study’s values were variable, we independently collected data depended on a valuation of how OSC and MIS affected WI and POACs in female CC patients [16, 17, 23].

### Study risk of bias assessment

To determine was there a possibility that any of the studies was biased, two authors individualistically reviewed the methodology of chosen examinations. “Risk of bias; RoB” instrument from the Cochrane Handbook for Systematic Reviews of Interventions Version 5.1.0 was utilized to estimate the bureaucratic quality. Following classification according to the judgement criteria, each research work was allocated to a specific bias risk category: low: If all quality criteria were adequately met, the research was categorized as having a low RoB. However, if any requirements were not met or not addressed, the study was classed as having a medium bias risk. The analysis identified a significant risk of bias if any of the quality requirements were not fully or partially completed.

### Effect measures

Only studies that evaluated and recorded the impact of open surgical management and MIS on WI and POACs in female CC patients were exposed to sensitivity analysis. Analysis of subclass was performed to assess sensitivity of females with CC and to compare OSC to MIS.

### Statistical analysis

The odds ratio (OR) with a 95% confidence interval (CI) were estimated utilizing dichotomous random- or fixed-effect models [24]. Calculated I [2] index has range of 0 to 100 and is expressed as a percentage [25]. Higher I [2] values signify increased heterogeneity, whilst lower I [2] values signify decreased heterogeneity. If I [2] was 50% or above, random effect was selected; otherwise, fixed effect was chosen [26]. First study’s consequences were classified as component of analysis of subcategory. Bias was measured by Begg’s and Egger’s tests for quantitative assessment, and it was considered to be existing if the estimated *P*-value was above 0.05 [27, 28]. *P*-values were calculated by the two-tailed method. With Jamovi 2.3, graphs and statistical analyses were created [29].

## Results

The study selected 44 papers published between 2002 and 2024 from all the related research that satisfied the inclusion criteria. Other studies were excluded for various reasons including studies that involved advanced stages of cervical cancer, patients who received chemotherapy prior to surgery, lacked unique data presentation, and articles that did not describe the outcome of interest. Table 2 displays the findings of these studies (30–72). Among the 11,631 female patients with CC who were at the beginning of the selected studies, 5072 were undergoing MIS, and 6559 were undergoing OSC. There were between 26 and 3333 females in the sample.

**Table 2:** Characteristics of the selected investigations for the meta-analysis

Study	Country	Study design	Total	Minimally invasive surgery			Open-surgical management			Outcomes measured
				No.	BMI (SD)	Age (years)	No.	BMI (SD)	Age (years)	
Lee, 2002 [30]	Taiwan	Prospective	60	30	54.4 (12.6)	46.2/	30	56.3 (10.4)	48	POACs
Steed, 2004 [31]	Canada	Retrospective	276	71	NA	43/	205	NA	44	POACs
Sharma, 2006 [32]	UK	Retrospective	67	35	NA	43.4	32	NA	42.8	POACs
Frumovitz, 2007 [33]	USA	Retrospective	89	35	28.1	40.8	54	28.2	42.5	POACs, PI & A
Li, 2007 [34]	China	Retrospective	125	90	NA	42	35	NA	44	POACs
Uccella, 2007 [35]	Italy	Retrospective	98	50	23	47	48	25	53	POACs
Morgan, 2007 [36]	Ireland	Retrospective	60	30	25	35	30	24	38	WI, POACs
Zakashansky, 2007 [37]	USA	Prospective	60	30	NA	48.3	30	NA	46.6	WI, POACs
Boggess, 2008 [38]	USA	Retrospective	100	51/ robotic laparoscopy	28.6 (7.2)	47.4	49	26.1	41.9	WI, POACs,, PI & A
Ko, 2008 [39]	USA	Retrospective	48	16/ robotic laparoscopy	27.6(6.4)	42.3	32	26.6 (5.9)	41.7	WI, POACs,, PI & A
Papacharalabous, 2009 [40]	UK	Retrospective	26	14	NA	38.6	12	NA	43.5	WI, POACs
Estape, 2009 [41]	USA	Retrospective	63	49/ robotic laparoscopy	29.7 (3.2)	55	14	28.1 (4.8)	52.8	POACs,, PI & A
Maggioni, 2009 [42]	USA	Retrospective	80	40/ robotic laparoscopy	24.1 (5.5)	44.1	40	23.6 (5.0)	49.8	WI, POACs
Malzoni, 2009 [43]	Italy	Retrospective	147	65	26	40.5	82	29	42.7	POACs
Sobiczewski, 2009 [44]	Poland	Retrospective	80	22	NA	45.4	58	NA	51.1	WI, POACs
Pahisa, 2010 [45]	Spain	Retrospective	90	67	25.4 (1.1)	51	23	27.2 2.5)	48	WI, POACs
Lee, 2011 [46]	Korea	Retrospective	72	24	23.4 (3.55)	48.4	48	23.9 (4.7)	50.2	POACs
Sert, 2011 [47]	Norway	Retrospective	68	42/ robotic laparoscopy	25.4 (4.36)	44.1	26	22.5 (1.84)	45	POACs
Taylor, 2011 [48]	USA	Retrospective	27	9	26.3	41.4	18	26.9	41.1	WI, POACs
Gortchev, 2012 [49]	Bulgaria	Retrospective	294	119	NA	46	175	NA	42.5	POACs
Park, 2012 [50]	Korea	Retrospective	166	54	31.8 (1.4)	49.4	112	31.7 (1.5)	52.1	WI, POACs
Nam, 2012 [51]	Korea	Retrospective	526	263	NA	NA	263	NA	NA	WI,, PI & A
Park, 2013 [52]	Korea	Retrospective	303	115	23.1	48.5	188	23.7	48.1	POACs,, PI & A
Lim, 2013 [53]	Singapore	Prospective	48	18	22.9	48	30	22.4	47	WI, POACs
Campos, 2013 [54]	Brazil	Randomized-controlled trial	30	16	NA	36.2	14	NA	39.6	POACs
Bogani, 2014 [55]	Italy	Retrospective	130	65	25.1 (5.2)	48.9	65	25.9 (6.1)	50.9	WI, POACs
Chen, 2014 [56]	Taiwan	Retrospective	100	56/ robotic laparoscopy	24.4 (4.9)	53.7	44	23.2 (3.4)	51.2	WI, POACs
Yin, 2014 [57]	China	Retrospective	45	22	NA	44	23	NA	46	WI, POACs
Asciutto, 2015 [58]	Sweden	Retrospective	250	65/ robotic laparoscopy	27.0 (6.0)	45.4	185	25.7 (4.7)	45.7	POACs
Xiao, 2015 [59]	China	Retrospective	154	106	23.8 (3.9)	43.7	48	24.7 (3.8)	45.7	WI, POACs
Ditto, 2015 [60]	Italy	Retrospective	120	60	24.4 (2.9)	46	60	24.0 (4.3)	45.5	POACs
Park, 2016 [61]	Korea	Retrospective	293	186	23.7	45.3	107	23.6	47.3	POACs
Shah, 2017 [62]	USA	Retrospective	311	109/ robotic laparoscopy	27.9	45.2	202	29.1	45.4	WI, POACs,, PI & A
Corrado, 2018 [63]	Italy	Retrospective	341	240/ robotic laparoscopy	23.3	46	101	23.5	45	WI, POACs
Guo, 2018 [64]	China	Retrospective	551	412	22.8	46	139	23.2	45	WI
Bogani, 2019 [65]	Italy	Retrospective	70	35	22.9 (4.0)	41.1	35	20.1 (9.3)	44.1	POACs
Matanes, 2019 [66]	Canada	Retrospective	98	74/ robotic laparoscopy	26.4	48	24	26.2	47	WI, POACs
Piedimonte, 2019 [67]	Canada	Retrospective	3333	749/ robotic laparoscopy	NA	NA	2584	NA	NA	WI, POACs
Yuan, 2019 [68]	China	Retrospective, single center	198	99	44.6 (7.6)	43.6	99	24.6 (1.5)	44.6	WI, POACs
Li, 2021 [69]	China	Retrospective	1207	661	NA	46.9	546	NA	47.03	POACs
Zaccarini, 2021 [70]	France	Retrospective	93	61	26.4 (4.7)	48.3	32	27.2 (6.0)	51	POACs,, PI & A
Jing, 2023 [71]	China	Retrospective	61	45	NA	49.06	16	NA	49.37	WI, POACs
Vázquez-Vicente a, 2024 [72]	Spain	Retrospective	117	39	25.4 (4.9)	47.1	78	25.6 (6.1)	46.8	WI, POACs
Vázquez-Vicente b, 2024 [72]	Spain	Retrospective	1156	633	25.1 (5.3)	46	523	25.7 (4.6)	48	WI, POACs
	**Total**		**11,631**	**5072**			**6559**			

NA: not available, PI & A: Pelvic infection & Abscess, POACs: postoperative aggregate complications, WI: wound infection

**Fig. 2:**
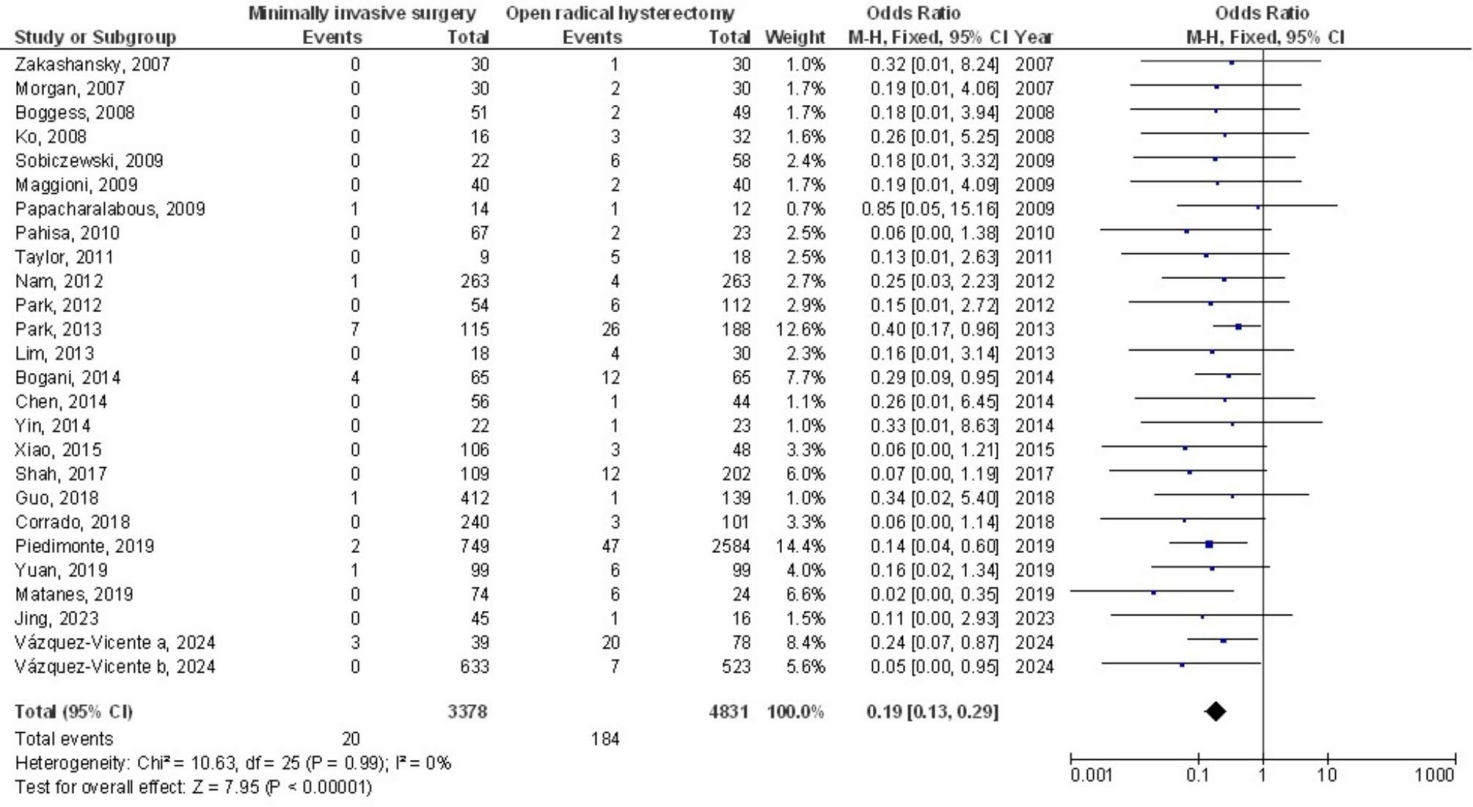
The forest plot analysis shows how wound infection in cervical cancer patients is affected by minimally invasive surgery as opposed to OSC

**Fig. 3:**
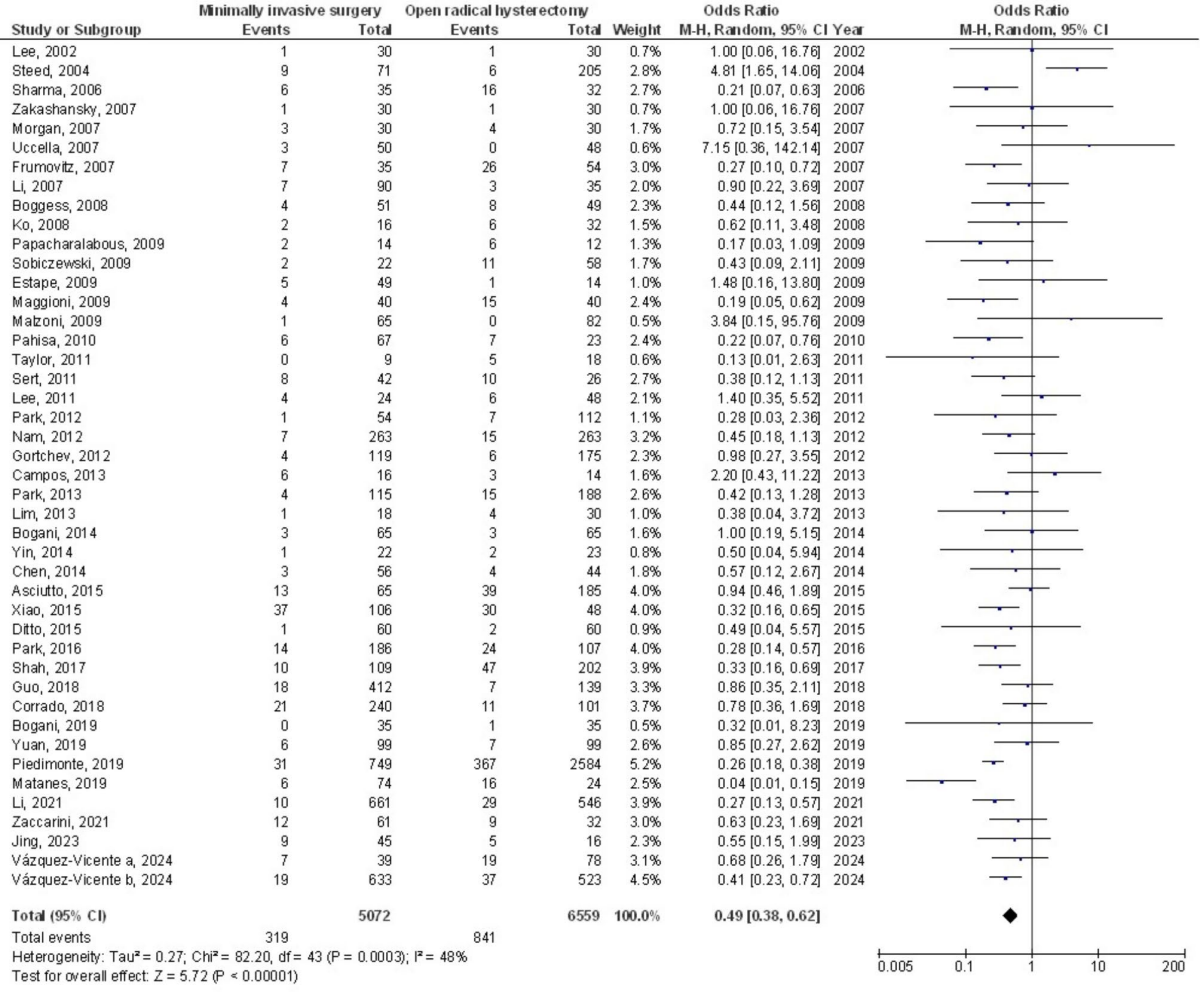
The forest plot analysis shows how POACs in cervical cancer patients were affected by minimally invasive surgery as opposed to OSC

**Fig. 4:**
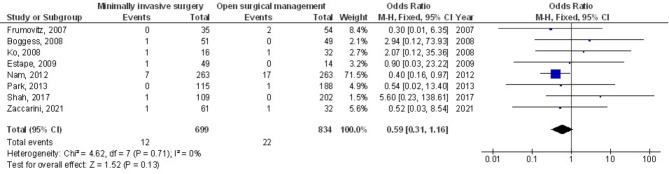
The forest plot of the minimally invasive surgery’s impact on PI&A in cervical cancer patients in comparison to OSC

Figures 2 and 3 show that, compared to open surgery, MIS had a much lower risk of WI (OR, 0.19; 95% CI, 0.13–0.29, *p* < 0.001) with no heterogeneity (I^2^ = 0%) and POACs (OR, 0.49; 95% CI, 0.38–0.62, *p* < 0.001) with moderate heterogeneity (I^2^ = 48%) in women with CC. Figure 4 shows that there wasn’t a considerable difference in PI&A between MIS and OSC for CC patients (OR, 0.59; 95% CI, 0.31–1.16, *p* = 0.13), and there was also no overlap (I^2^ = 0%).

Subgroup analysis of studies based on the use of MIS techniques with or without robotics showed a consistently significant effect size for wound infection outcome [(OR, 0.23; 95% CI, 0.15–0.37, *p* < 0.001), and (OR, 0.11; 95% CI, 0.05–0.28, *p* < 0.001), respectively] with no heterogeneity between studies (I^2^ = 0%). Similarly, POACs subgroup analysis based on robotics use in laparoscopic procedure revealed consistent significance [(OR, 0.54; 95% CI, 0.41–0.71, *p* < 0.001, I^2^ = 34%) without robotics, and (OR, 0.38; 95% CI, 0.23–0.62, *p* < 0.001, I^2^ = 66%).) with robotics use], respectively. The effect size for both outcomes was smaller for robotic-based laparoscopy procedures versus without robotics.

Subgroup analysis based on robotic-based laparoscopy for the PI &A outcome showed a significant effect with the conventional laparoscopy (OR, 0.40; 95% CI, 0.18–0.89, *p* = 0.02, I^2^ = 0%), while robotic based procedures resulted in non-significant pooled estimate (OR, 2.42; 95% CI, 0.51–11.41, *p* = 0.26, I^2^ = 0%).

The visual interpretation of the forest plot effect and the quantitative Egger regression test revealed no indication of investigation bias (*p* = 0.890). The findings revealed that the majority of relevant examinations lacked practical quality and exhibited bias in their selective reporting.

## Discussion

Of the studies that were considered for the meta-analysis, 5072 of the 11,631 females with CC who were at baseline of the selected investigations were using MIS, and 6559 were using open surgical management [30–72]. When compared to OSC, women with CC who underwent MIS had significantly fewer WI and POACs, but no discernible difference in PI&A. These findings are in accordance with the results of meta-analyses conducted by Kampers et al., [73], Zhang et al. [74], and Zhao et al. [75] which compared MIS versus OSC confirming the significant efficacy and safety of MIS with similar non-significance in the postoperative infection and abscess formation compared to OSC in the general and subgroup analysis. It is noteworthy that our results only included eight relevant studies in the PI&A outcome. Our analysis may be biased due to a limited number of researches comparing PI & A outcome between the two surgical techniques.

The small sample size of some of the chosen examinations (23 out of 44 ≤ 100 females) for meta-analysis requires caution when interpreting their results. Limited patient samples may introduce bias contingent upon the surgeon’s proficiency in novel surgical procedures, particularly robotic hysterectomy. This would impact how significant the evaluated assessments were [76–86]. Therefore, randomized studies with larger sample size are needed to better validate the current evidence.

The use of Veress needles throughout the process might account for the dissimilarities in abdominal damage between MIS and open surgical management [87]. The Veress needle methodology is a contained method that entails the placement and retracting of a sharp-tipped 2-mm external needle, succeeded by a hollow blunt-tipped needle that advances to provide gas. Insufflation at different pressure, duration, or volume parameters happens before to the placement of the main trocar. This technique is the predominant entry method employed by gynecologists globally, and is associated with heightened chances of mild problems, such as preperitoneal injuries, as well as entry failure [88]. The 2012 Cochrane database determined that the open entry technique significantly reduces the rate of failed entry as opposed to the closed entry technique, without variation in the occurrence of visceral or vascular harm. The minimal incidence of reported complications linked to laparoscopic entry and the limited participant pool in the trials may explain the absence of a substantial disparity in major visceral and vascular injuries between the entry procedures [89]. Previous studies have shown that a Veress needle or trocar entrenched throughout laparoscopy causes most intestinal damage and WIs. A number of risk factors could produce subcutaneous emphysema in MIS [90, 91]. The skill of the surgeon could affect the frequency of complications when considered holistically. Regretfully, this study was unable to compare different surgeons. When comparing various surgical methods, the learning curve may also significantly impact issues. MIS gained preferred over open surgical care because to the problems of the surgical approach [92, 93]. Utilization of surgical tools has been connected to injuries, which could be the consequence of thermal injury because the elevated temperature of the tools harms the deeper or sub-mucosal tissues of the gut, bladder, and bowels. Previous studies have assessed the heat damage to the intestines caused by laparoscopic surgery [90]. Recall that the open surgical treatment approach raised the risk of heat injury, so surgeons needed to be attentive to this. All of these variables were associated with the development of WIs and POACs. This aligns with the results of the former meta-analyses. Refinement of laparoscopic-assisted vaginal RH is crucial because of the intricate nature of the female pelvic floor. Urinary tract injuries are a severe problem with MIS. The uterine ligament is identified and removed in the vaginal approach, which next involves pushing on the uterus to find the bladder and ureters [94]. The gradual drop in PI&A can be ascribed to the emergence of laparoscopy as a result of advances in surgical methods, equipment, and learning curves. Compared to OSC, complete laparoscopes and robotic RHs were linked with a lower risk of POACs [92]. An earlier study by Park et al., which investigated the unfavorable impacts of the three treatments, supported these results by indicating that MIS was superior than OSC in terms of minimizing overall difficulties for females with CC [10]. The results on POACs for the group that underwent open surgical management-aided vaginal RH might be biased because of the significant heterogeneity degree and small sample size. In the future, further high-quality cohort investigations will be required to compare and estimate the risk of POACs in MIS and OSC.

Eleven studies included robotic-based laparoscopy versus OSC. We compared both robotic and non-robotic laparoscopy to OSC. The results showed that robotic-based procedures had a significant but smaller effect size on the WI and POACs outcomes. These findings are consistent with a recently published meta-analysis, which reported that conventional laparoscopic procedures have a much lower operating time and overall complication rate. Robotic laparoscopy did not improve treatment outcomes, but its application did reduce blood loss [95].

This meta-analysis validated the effects of OSC and MIS on WI and CC control. Based on the current meta-analysis findings, MIS procedures can be a preferred alternative for open surgical procedures with better outcomes in terms of wound infection and the overall postoperative morbidities. Moreover, the surgeon’s skills and proficiency may influence the incidence of complications following the procedure. Further investigation is still needed to elucidate these plausible influences. This was also highlighted in earlier studies that generated equal impact levels through the use of a correlated meta-analysis technique [96–103]. Well-led RCTs are crucial to take into account these aspects and the diversity of dissimilar ages and demographics of female participants, even if meta-analysis was unable to establish whether modifications in these features are associated to the values being studied. In conclusion, among female patients with CC, OSC resulted in dramatically decreased WIs as compared to MIS.

### Limitations

Possible selection bias may have been present due to the exclusion of some studies in the meta-analysis. However, the publications that were excluded did not match the requirements to be included in the meta-analysis. However, we required the data in order to assess if demographic and age disparities had an impact on the outcomes. The objective of the exploration was to assess the influence of open surgical management and minimally invasive surgery (MIS) on wound infection (WI) and complication rate (CC) in the treatment. The presence of inaccurate or missing data in previous studies may have contributed to an increased bias. Aside from their age and race, the nutritional well-being of the girls was a possible factor contributing to discrimination. Inadequate data and unpublished investigations can lead to unwanted distortion of the value being examined.

## Conclusions

When compared to OSC, MIS resulted in much lower WI and POACs; however, there was no discernible difference in PI&A rates among female patients with CC. However, the small sample size of several specified investigations (23 out of 44 ≤ 100 female patients) necessitates caution when interpreting the data in the meta-analysis, nevertheless. That would have an impact on how significant the evaluated assessments were.

## Electronic Supplementary Material

Below is the link to the electronic supplementary material.

Supplementary Material 1

## Data Availability

On request, the corresponding author is required to provide access to the meta-analysis database.
